# Infusing Silicone and Camellia Seed Oils into Micro-/Nanostructures for Developing Novel Anti-Icing/Frosting Surfaces for Food Freezing Applications

**DOI:** 10.1021/acsami.3c02342

**Published:** 2023-03-10

**Authors:** Zhiwei Zhu, Hui Liang, Da-Wen Sun

**Affiliations:** † School of Food Science and Engineering, South China University of Technology, Guangzhou 510641, China; ‡ Academy of Contemporary Food Engineering, South China University of Technology, Guangzhou Higher Education Mega Center, Guangzhou 510006, China; § Engineering and Technological Research Centre of Guangdong Province on Intelligent Sensing and Process Control of Cold Chain Foods, & Guangdong Province Engineering Laboratory for Intelligent Cold Chain Logistics Equipment for Agricultural Products, Guangzhou Higher Education Mega Centre, Guangzhou 510006, China; ∥ Food Refrigeration and Computerized Food Technology (FRCFT), Agriculture and Food Science Centre, University College Dublin, National University of Ireland, Belfield, Dublin 4, Ireland

**Keywords:** slippery liquid-infused porous surface, superhydrophobic surface, frosting delay, ice adhesion strength, food processing

## Abstract

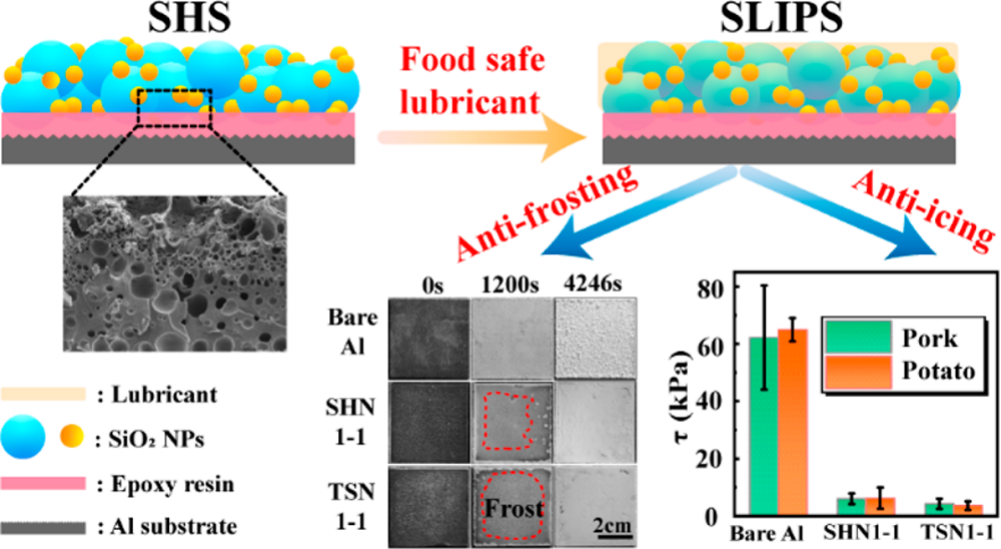

Undesired ice/frost formation and accretion often occur on food freezing facility surfaces, lowering freezing efficiency. In the current study, two slippery liquid-infused porous surfaces (SLIPS) were fabricated by spraying hexadecyltrimethoxysilane (HDTMS) and stearic acid (SA)-modified SiO_2_ nanoparticles (NPs) suspensions, separately onto aluminum (Al) substrates coated with epoxy resin to obtain two superhydrophobic surfaces (SHS), and then infusing food-safe silicone and camellia seed oils into the SHS, respectively, achieving anti-frosting/icing performance. In comparison with bare Al, SLIPS not only exhibited excellent frost resistance and defrost properties but also showed ice adhesion strength much lower than that of SHS. In addition, pork and potato were frozen on SLIPS, showing an extremely low adhesion strength of <10 kPa, and after 10 icing/deicing cycles, the final ice adhesion strength of 29.07 kPa was still much lower than that of SHS (112.13 kPa). Therefore, the SLIPS showed great potential for developing into robust anti-icing/frosting materials for the freezing industry.

## 1 Introduction

Undesired ice/frost formation and accretion often occur on food freezing facility surfaces,^1,2^ which reduces heat transfer efficiency on evaporators and decreases cooling capacity by up to 20% in refrigeration systems.^3−5^ Therefore, various defrosting methods^6−8^ have been studied. Traditional common deicing/frosting methods include compressor shutdown, using hot gas, hot water or electric heating, and solvent defrosting, which have low efficiency and high energy consumption and can easily cause mechanical damage.^9^ Besides, frequent defrosting can cause temperature fluctuations,^10^ leading to negative effects on food quality.

In order to overcome the above disadvantages associated with the traditional deicing/frosting methods, superwetting strategies have been investigated. Through surface modification to delay the water-to-ice phase transition and reduce the adhesion between ice and facility surfaces, these superwetting strategies can provide passive anti-icing performance,^11−13^ which ultimately reduces icing/frosting hazards and energy consumption of active defrosting.^14,15^ Barthlott and Neinhuis designed and fabricated superhydrophobic surfaces (SHS) based on the mechanism of water-repellent lotus leaf,^16^ forming water droplets in the Cassie–Baxter state on the surface due to the micro-/nanoscale structure with a low surface energy,^17^ and the ice phobic mechanisms and applications of SHS have recently attracted much research attention due to its superior water repellency and icing delay ability.^18−21^ However, the infiltration of condensate droplets into the air pocket on anti-icing/frosting SHS in high humidity environments has also been reported, resulting in the formation of strong mechanical interlocking between ice and the rough structure of the surface, further increasing ice adhesion.^22^ In addition, Chen et al. also reported that the ice adhesion strength increased linearly with the increase of the area fraction of air in contact with liquid.^23^

To solve the above problems of SHS, slippery liquid-infused porous surfaces (SLIPS) have been developed,^24^ in which a continuous smooth lubricant is injected into the micro-/nanostructure to achieve extremely low ice adhesion.^13^ Long et al. fabricated SLIPS by infusing silicone oil into porous SHS, which exhibited excellent liquid repellency, anti-corrosion, and anti-icing performances.^25^ To further enhance the mechanical properties of SLIPS, Tan et al. developed a SLIPS with micro pyramidal holes (P-SLIPS) with only 11.5 kPa ice adhesion obtained by injecting perfluoropolyether (PFPE) lubricant into anisotropic etching and spraying SHS coating.^26^ In addition, by tailoring the cross-link density of different elastomeric coatings and by additionally embedding miscible polymeric chains, Golovin et al. systematically designed interfacial slippage ice phobic surfaces in smooth and nonporous coatings.^27^ However, most of the ice phobic surfaces in these studies (Table 1) lack durability tests,^28−30^ and other studies either were conducted with only a few icing/deicing cycles or showed poor results after cycling.^31−38^ More importantly, materials having fluorocarbon bonds are always used as the candidates for the fabrication of SHS and SLIPS due to their excellent low surface energy;^21,39,40^ however, these materials often degrade to perfluorooctanesulfonate (PFOS), which is a persistent metabolite that accumulates in tissues of humans,^41,42^ causing potentially harmful influence to human health, and thus significantly limiting their applications in food processing facilities and food packaging.

**Table 1:** Summary of Measured Multiple Icing/Deicing Cycles of SHN1-1 and TSN1-1 in Comparison with Results Reported in the Literature for Ice Phobic SLIPS^a^

			ice-adhesion strength (kPa) of each icing/deicing cycle										
coatings	lubricant	testing method	1	2	3	4	5	6	7	8	9	10	reference
SHN1-1	silicone oil	plastic cuvettes icicle test (10 mm × 10 mm ×45 mm)	6	12	15	14	18	19	21	22	23	29	this work
TSN1-1	camellia seed oil	plastic cuvettes icicle test (10 mm × 10 mm ×45 mm)	14	16	23	24	27	29	34	33	34	37	
PDMS	PDMS-PEG copolymers	plastic cuvettes icicle test (10 mm × 10 mm ×45 mm)	59	61	58	-	-	-	-	-	-	-	(31)
LC-20-0	silicone oil	glass column with cross-sectional area of π × 9^2^ mm^2^	19	16	20	-	-	-	-	-	-	-	(32)
SLI-EG	ethylene glycol	bottomless glass square cuvette	<1	3	21	-	-	-	-	-	-	-	(33)
PTFE	perfluorinated oil	centrifugal ice adhesion test	8	8	13	10	-	-	-	-	-	-	(34)
PTFE	silicone oil	centrifugal ice adhesion test	13	17	22	21	-	-	-	-	-	-	
PP	silicone oil	centrifugal ice adhesion test	33	59	60	62	-	-	-	-	-	-	
SHC	silicone oil	plastic cuvettes icicle test (10 mm × 10 mm ×45 mm)	<1	5	10	6	4	3	3	-	-	-	(35)
SFC	perfluoropolyether	Plastic cuvettes icicle test (10 mm × 10 mm ×45 mm)	<1	2	4	2	1	<1	<1	-	-	-	
SLIPNS	silicone oil	cuboid ice blocks (contact area *A* = 10 × 10 mm^2^)	48	48	-	49	-	52	-	53	-	54	(36)
LIC3	fully hydrogenated cottonseed oil	centrifugal ice adhesion test	23	28	40	63	-	-	-	-	-	-	(37)
LIMNSMC	silicone oil	hollow cylinders	19	20	8	16	13	25	61	75	73	86	(38)

a Note: “-” indicates no relevant test. PDMS, polydimethylsiloxane; PDMS-PEG copolymers, polydimethylsiloxane poly(ethylene glycol) copolymers; LC-20-0, lubricant-infused coating, 20 and 0 are the weight percentages of silicone oil and SiO_2_ NPs in lubricant-infused coating solution, respectively; SLI-EG, slippery surfaces transfused with ethylene glycol; PTFE, polytetrafluoroethylene; PP, polypropylene; SHC, slippery hybrid coating; SFC, fluorous slippery coating; SLIPNS, slippery liquid-infused porous network surface; LIC3, lubricated icephobic coating; LIMNSMC, liquid-infused micro-nanostructured MOF coatings.

Therefore, the current study aimed to design inexpensive and scalable fluorine-free SLIPS by infusing two kinds of food-grade lubricants into micro-/nanostructures constructed with modified dual-scale SiO_2_ NPs and epoxy resin. The icephobicity of these two SLIPS was evaluated by determining their frosting delay and the adhesion strengths of ice and food (pork and potato). In addition, multiple icing/deicing cycles were carried out to examine their durability. It is hoped that results from the current study should shed more light on developing robust anti-icing/frosting materials for applications in food freezing and storage facilities.

## 2 Materials and Methods

### 2.1 Materials

SiO_2_ NPs with sizes of 15 ± 5 and 50 ± 5 nm, stearic acid (SA, 98%), and silicone oil were obtained from Macklin Reagent Co., Ltd. (Shanghai, China). Hexadecyltrimethoxysilane (HDTMS, 97%) was supplied by Shanghai Bide Medical Technology Co., Ltd. (Shanghai, China). Camellia seed oil was provided by Guangzhou Yiyikoutian Organic Agriculture Co., Ltd. (Guangzhou, China). Tetraethyl orthosilicate (TEOS, 99.9%), E44 epoxy resin with a curing agent, anhydrous ethanol, hydrochloric acid (HCl), and acetone were acquired from Aladdin Reagent Co., Ltd. (Shanghai, China). Fresh pork and potato were purchased from a local market (Guangzhou, China). All these reagents were used without further purification. Aluminum (Al) sheets with sizes of 45 × 45 × 1 mm^3^ were bought from Hongwang Mold Co., Ltd. (Shenzhen, China) and used as the substrate. In addition, deionized water made by Direct-Pure UP UV 10 (RephiLe Bioscience, Ltd., Zhejiang, China) was used throughout the work.

### 2.2 Fabrication of SHS and SLIPS

#### 2.2.1 Preparation of HDTMS-Modified SiO_2_ Suspensions

The SHS was prepared according to Li et al. with some modifications.^43^ First, 0.3 g of dual-sized SiO_2_ NPs (15 ± 5 and 50 ± 5 nm, 1:1) was ultrasonically (SB-600DTY, Scientz Inc., Ningbo, China) dispersed in an HCl aqueous solution (0.01 M, 50 mL) for 20 min. Subsequently, 0.5 g of HDTMS and 0.1 g of TEOS were added dropwise into the mixture, which was further vigorously stirred using a magnetic stirrer (C-MAG HS10, IKA GmbH, Staufen, Germany) at 800 rpm for 24 h under a room temperature of 25 °C, forming a uniform HDTMS-modified SiO_2_ NPs suspension. After drying in an oven (DHG-9240A, Shanghai Bluepard Instruments Co., Ltd., Shanghai, China) at 60 °C for 12 h, the HDTMS-modified SiO_2_ NPs were obtained and designated as H-SiO_2_ NPs.

#### 2.2.2 Preparation of SA-Modified SiO_2_ Suspensions

Following the method of Daneshmand et al.,^44^ 0.1 g of SA was first ultrasonicated in 10 mL of anhydrous ethanol for 10 min. Then, 0.3 g of dual-sized SiO_2_ NPs (15 ± 5 and 50 ± 5 nm, 1:1) was dispersed into the above ethanol solution and ultrasonicated for 1 h to react adequately. After drying in the oven at 60 °C for 12 h, the SA-modified SiO_2_ NPs were obtained and designated as S-SiO_2_ NPs, which were further ultrasonically dispersed in HCl aqueous solution (0.01 M, 50 mL) for 30 min and magnetically stirred at 800 rpm for 2 h under room temperature to obtain the uniform S-SiO_2_ NPs suspensions.

#### 2.2.3 Preparation of SHS and SLIPS

For preparing SHS, Al substrates previously ground with 180 mesh sandpapers were ultrasonically cleaned using 0.1 M HCl, 0.1 M acetone, anhydrous ethanol, and deionized water sequentially for 10 min and dried in the oven at 60 °C for 4 h before use. E44 epoxy resin and its curing agent (2:1) were ultrasonically dissolved in a moderate amount of anhydrous ethanol. One milliliter of mixed epoxy resin solutions was a brushed coating on the pretreated Al substrates, and 4.0 mL of H-SiO_2_ NPs suspensions or S-SiO_2_ NPs suspensions were sprayed (R2-F, Prona Air Tool Manufacturing Ltd., Taiwan, China) onto the semicuring epoxy resin coating. After curing completely under room temperature for 24 h, two SHS were obtained and named HN1-1 and SN1-1, respectively.

**Figure 1:**
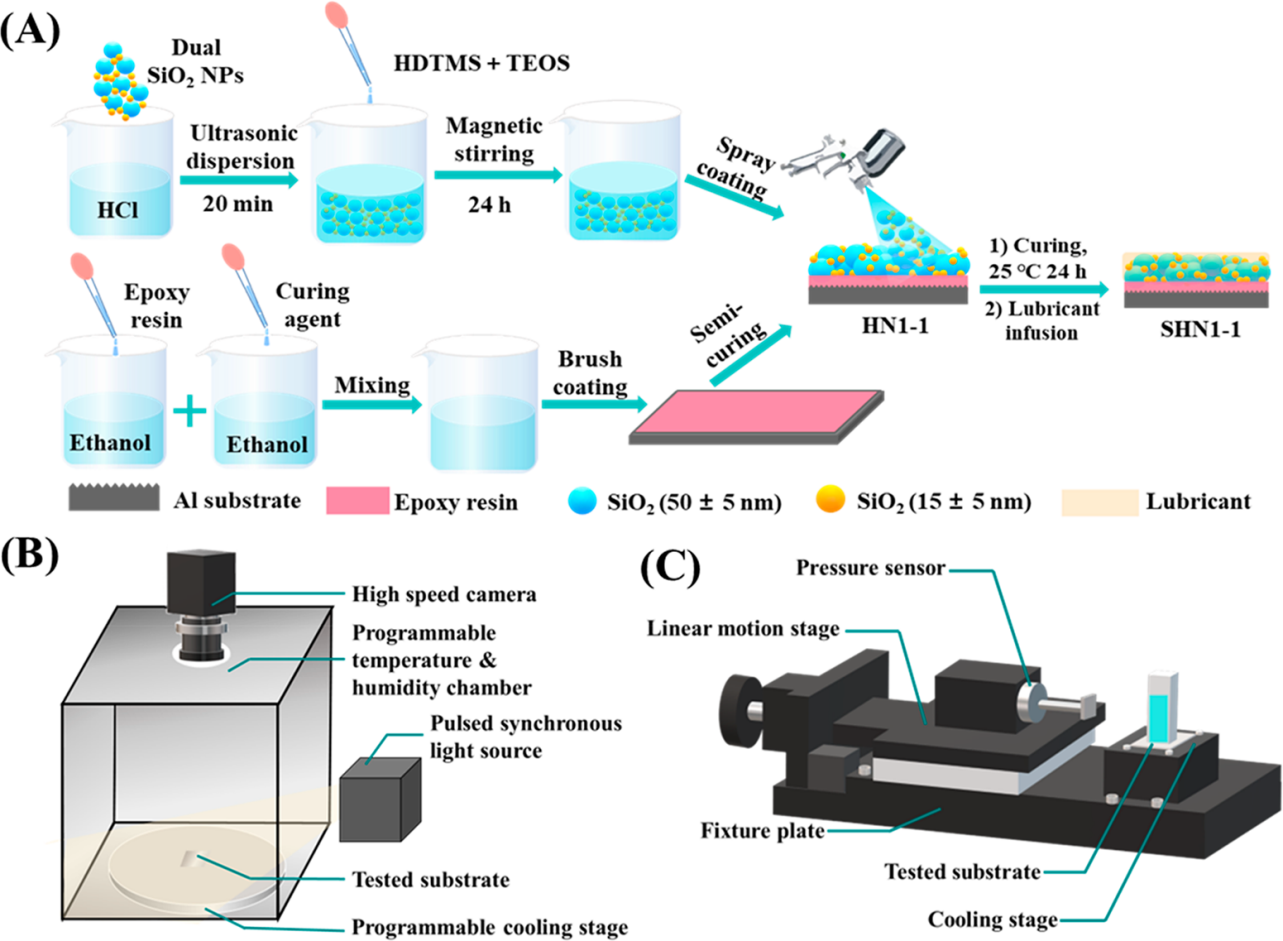
(A) Schematic diagram of preparation process of H-SiO_2_ NPs SHS and SLIPS surface. Schematic diagrams and the main working parts of devices for (B) anti-frosting test and (C) the ice adhesion measurement.

The preparation of SLIPS was according to the method of Long et al.^25^ The micro-/nanoscale porous structures of HN1-1 and SN1-1 then were infused with approximately 200.0 μL of silicone oil and camellia seed oil, separately, to obtain two SLIPS and designated as SHN1-1 and TSN1-1, respectively. All samples were placed vertically for 30 min at room temperature to remove excess liquid after being fully lubricated. The preparation processes of HN1-1 and SHN1-1 are illustrated in Figure 1A, and the detailed amount of each component and corresponding sample ID are shown in Table 2.

**Table 2:** Sample ID and the Corresponding Weight Ratio of the Two Types of SLIPS with Different SiO_2_ NPs Sizes

		SiO_2_ NPs (g)		modifier (g)				lubricant (μL)	
sample ID	0.1 M HCl (mL)	15 ± 5 nm	50 ± 5 nm	HDTMS	SA	epoxy resin (g)	curing agent (g)	silicone oil	camellia seed oil
SHN1-1	50	0.15	0.15	0.5	0	3	1.5	200	0
TSN1-1	50	0.15	0.15	0	0.1	3	1.5	0	200

### 2.3 Characterization

#### 2.3.1 Chemical Structure and Surface Topography

Fourier transform infrared (FTIR) spectra ranging from 4000 to 500 cm^–1^ were acquired using an FTIR spectrometer (Tensor 27, Bruker Inc., Karlsruhe, Germany), and the samples were tested by the potassium bromide pellet method. The surface topography of the prepared samples was determined using a 3D optical profilometer (Up-Dual Model, Rtec Instruments Inc., San Jose, CA) with a scanning area of 0.66 mm × 0.88 mm, and the arithmetic mean surface roughness (*S*_a_) was calculated from the scanned images, using the following equation:^45^
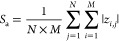
1where *M* and *N* refer to the number of points sampled in the lateral *x* and *y* directions, respectively, and *z* means the vertical distance of a certain point to the mean plane of the surface.

To verify the aggregation of SiO_2_ NPs on the epoxy resin, the HN1-1 with a thin gold layer was captured by a scanning electron microscopy (SEM) analysis (MERLIN, Carl Zeiss Microscopy GmbH, Jena, Germany). The thicknesses of the HN1-1 and SN1-1 were measured by capturing the cross-sectional images using SEM, and 15 parallel tests were proceeded to obtain reliable values.

#### 2.3.2 Surface Wettability

The wettability of the fabricated samples was characterized by measuring the static contact angle (CA) at room temperature, using an optical contact angle goniometer (OCA40 Micro, Dataphysics Instruments GmbH, Filderstadt, Germany), which adopted the sessile drop method by dropping 6.0 μL of deionized water.

#### 2.3.3 Anti-Frosting Property

The anti-frosting property was performed using a programmable temperature and humidity chamber (DJL-HW80, Dejieli Co., Ltd., Shenzhen, China) equipped with a pulsed synchronous light source (FS 100, Rongfeng Photoelectric Technology Co., Ltd., Guangzhou, China) (Figure 1B), which was previously reported.^46^ The prepared samples were directly placed on the cooling stage with an ambient temperature of 5 °C, a cooling stage temperature of −15 °C, and relative humidity of 55% as the frosting conditions and the videos of the sample surface were captured by a high-speed digital camera (AT-X M100 f/2.8 PRO D Macro, Kenko Tokina Co., Ltd.) at 50 frames per second until the surfaces of samples were completely frosted. Besides, the initial mass of the prepared samples was weighed in advance, and then, each sample and the frost that formed on it were weighed every 30 min during frosting, and the difference between the two weights was defined as the frosting quantity. After being frozen under −15 °C for 3 h, the samples were placed vertically at room temperature of 20 °C for observing the natural defrosting process with the high-speed digital camera.

#### 2.3.4 Ice Phobic Property

The ice phobic property of the SHS and SLIPS was evaluated by ice adhesion strength (τ_ice_), which was measured using a previously reported device,^46^ as shown in Figure 1C. It was mainly composed of a pressure sensor (SBT673, SIMBATOUCH Co., Ltd., Guangzhou, China), a linear motion stage (NT101TA75M, NATE-Optics Inc., Langfang, China), and a cooling stage (XH-X251, XINGHE Electronics Co., Ltd., Suzhou, China). The prepared samples were placed on the cooling stage, and plastic cuvettes (10 mm × 10 mm × 45 mm) were inverted on sample surfaces. Approximately 2.0 mL of deionized water was injected into the cuvettes through a premade hole at the top. After being frozen under −15 °C for 3 h, the ice adhesion test was performed by setting the linear motion speed as 1.0 mm s^–1^ and trigger force as 0.05 N. The equation of ice adhesion strength (kPa) was defined as:^21^
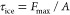
2where *F*_max_ is the measured ice adhesion force (N) and *A* is the cross-sectional contact area of ice and the surface (cm^2^).

### 2.4 Statistical Analysis

Three parallel tests were carried out for each measurement unless stated otherwise, and the obtained data were expressed as mean ± standard deviations. The videos captured from the high-speed digital camera were processed by TroublePix (Norpix Inc., Montreal, Canada). One-way variance analysis using Tukey’s test at significant levels of *p* < 0.05 was performed using SPSS 18.0 (SPSS Inc., Chicago, USA).

## 3 Results and Discussion

### 3.1 Fabrication of the SHS and SLIPS

**Figure 2:**
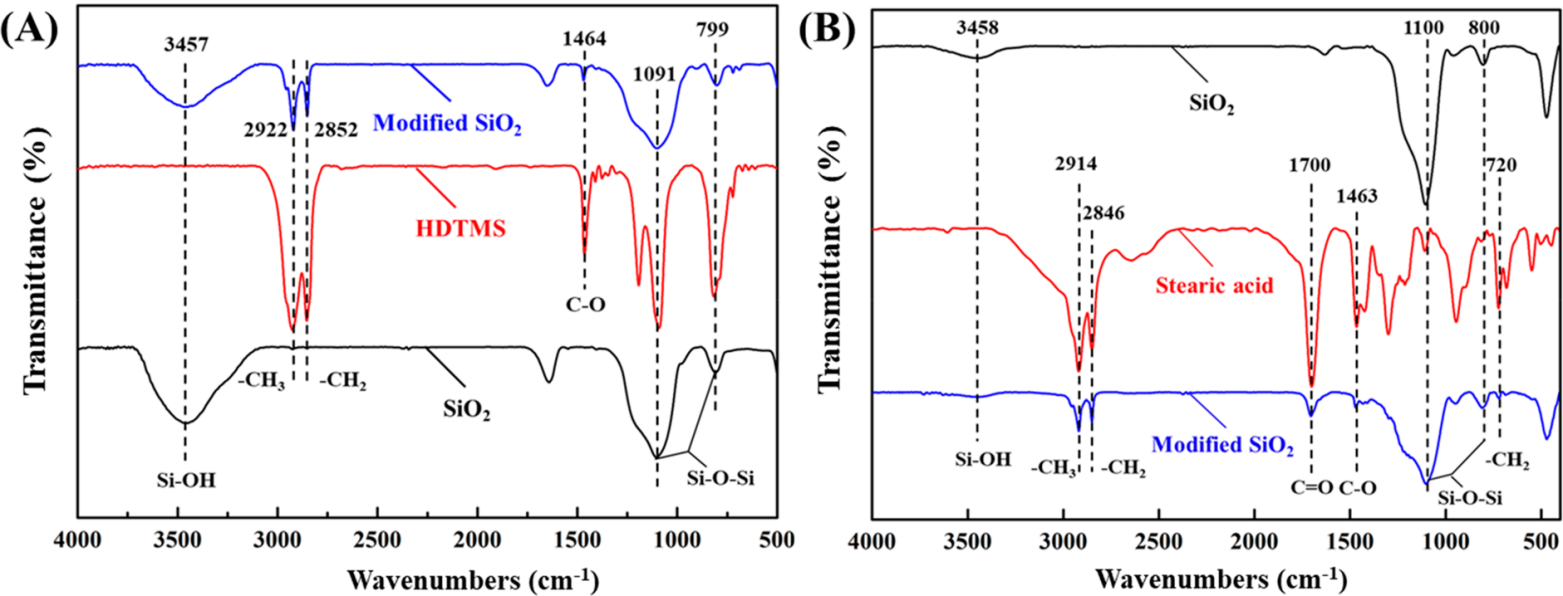
FTIR spectra of SiO_2_ NPs before and after (A) HDTMS and (B) SA modification.

The surface chemical structures of SiO_2_, H-SiO_2_ NPs, and S-SiO_2_ NPs were examined according to the FTIR spectra shown in Figure 2. Compared with the raw SiO_2_ NPs, these characteristic absorption bands of the H-SiO_2_ NPs at 3457 cm^–1^ (corresponding to −OH stretching vibration),^47^ 1091 cm^–1^ (belonging to asymmetric stretching vibrations of Si–O–Si) and 799 cm^–1^ (attributed to Si–O–Si bending mode)^21^ were markedly enhanced. The new band at 1464 cm^–1^ was generated owing to the C–O stretching vibration groups, showing that the Si–OH groups were covalently bonded to the hexadecyl groups of the HDTMS. More importantly, two new bands at 2922 and 2852 cm^–1^ were detected (Figure 2A), which were ascribed to the stretching vibrations of −CH_3_ and −CH_2_ bands in HDTMS.^43^ Similarly, the absorption bands in Figure 2B showed that the SiO_2_ NPs were also successfully modified by SA. Especially, the absorption peak at 1700 cm^–1^ was the carbonyl (C=O stretching) according to the FTIR spectrum of S-SiO_2_, demonstrating the destabilization of the double bond in the carbonyl and interaction with −OH group on the surface of SiO_2_.^44^ The above results demonstrated the successful grafting of low-surface-energy groups on the SiO_2_ NPs surface.

**Figure 3:**
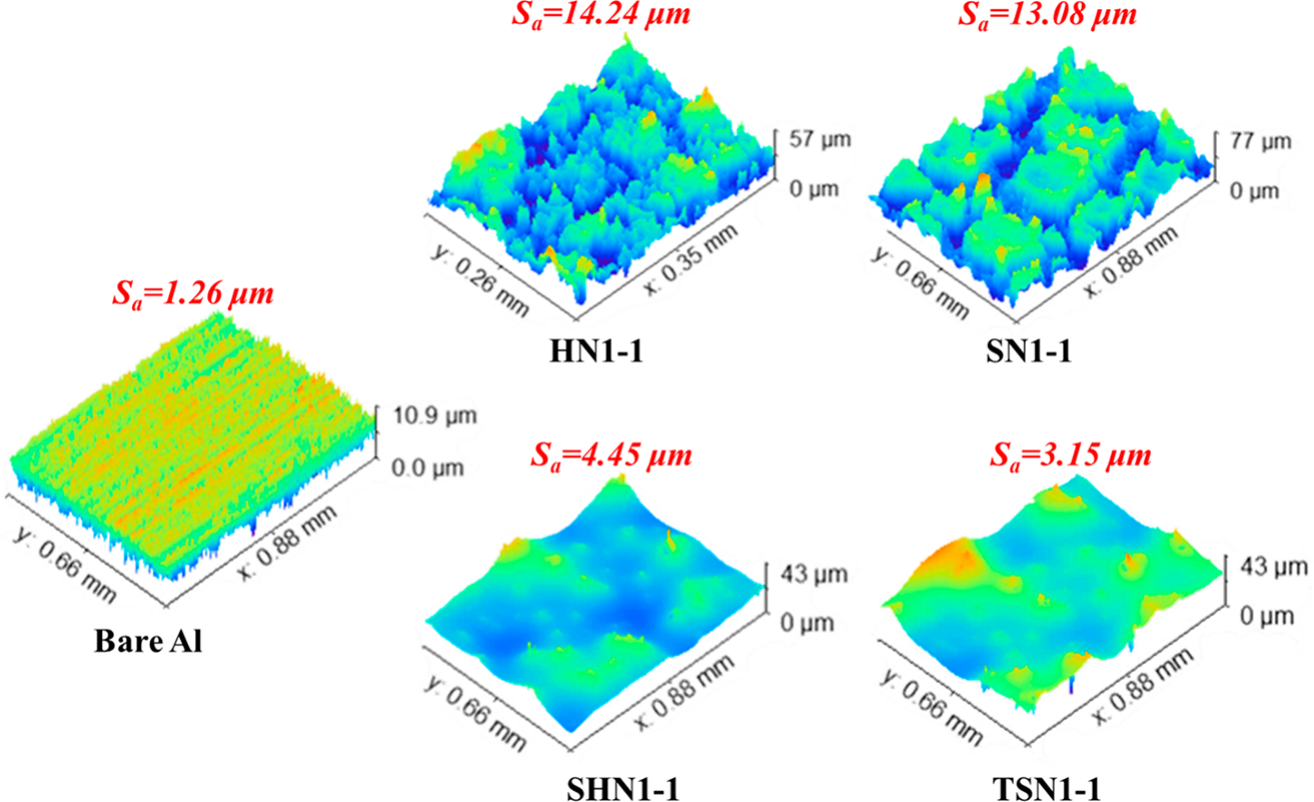
3D surface profiles and *S*_a_ values of prepared SHS and SLIPS.

**Figure 4:**
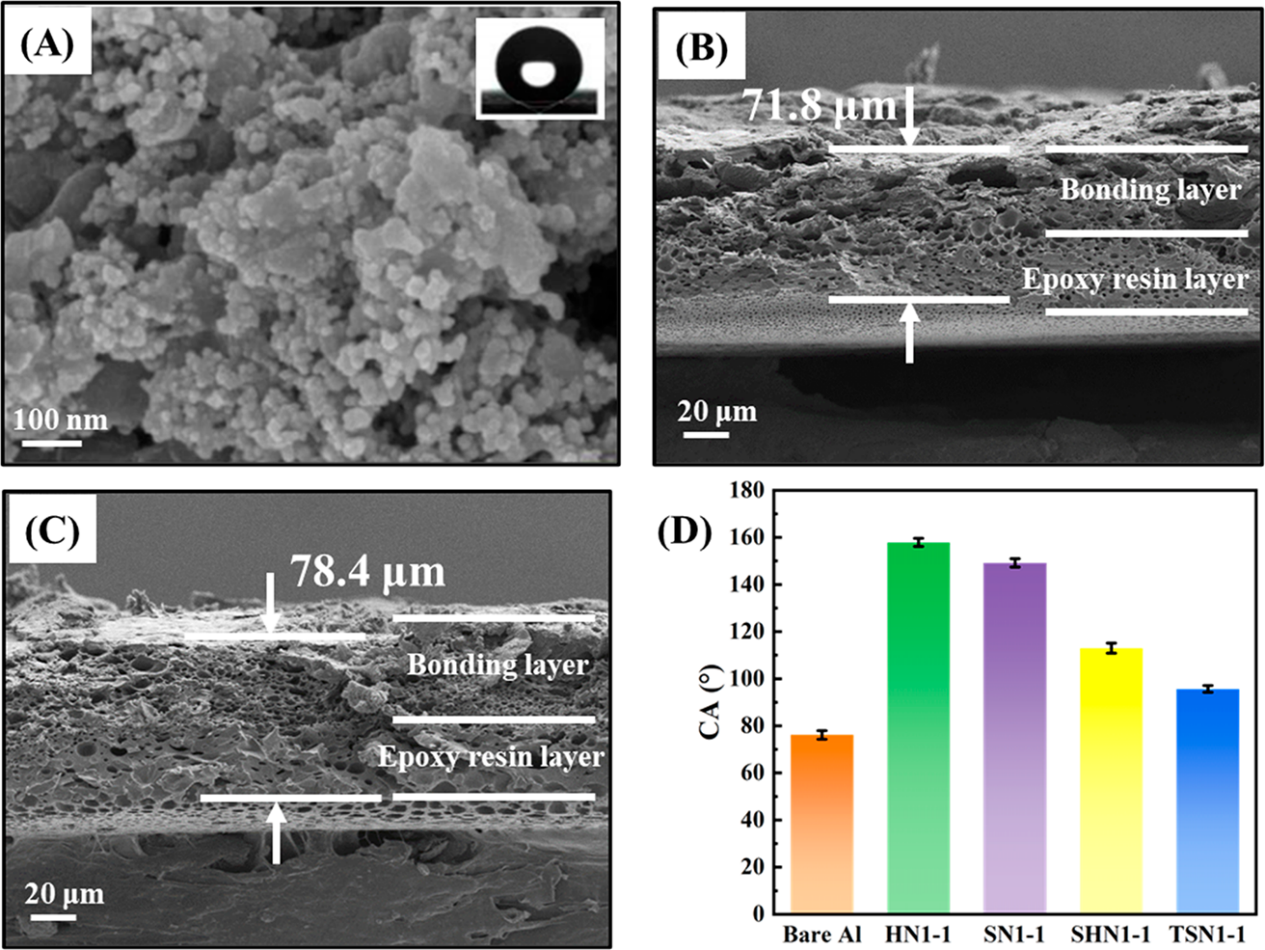
(A) SEM surface topography of HN1-1 with 100 000 times magnification, with the inset being 6 μL droplets for measuring CA on it. SEM images of the cross sections of the (B) HN1-1 and (C) SN1-1. (D) CA of bare Al and prepared SHS and SLIPS.

The surface morphologies of the SHS and SLIPS were investigated by 3D surface profile. The surface of bare Al was uniform and smooth with a *S*_a_ of 1.26 μm (Figure 3), while obvious “peaks” and “valleys” composed of dual scale SiO_2_ NPs could be seen on the surfaces of HN1-1 and SN1-1 with much greater *S*_a_ values of 14.24 and 13.08 μm, respectively. The space between conglobated dual-scale SiO_2_ NPs was vital to trap air in the “pockets”, showing a significant sizing effect of micro-/nanostructures on SHS (Figure 4A). Meanwhile, SEM images of the cross sections clearly showed that the thicknesses of HN1-1 and SN1-1 were 71.8 and 78.4 μm, respectively (Figure 4B,C). The coatings mainly consisted of an epoxy resin layer and its bonding layer with a modified SiO_2_ layer, showing a distinct porous structure, which was conducive to the penetration and retention of lubricants. After being infused with silicone oil and camellia seed oil, the SHN1-1 and TSN1-1 surfaces became relatively smooth (Figure 3) with their *S*_a_ significantly dropped down to 4.45 and 3.152 μm, respectively, indicating that the silicone oil and camellia seed oil could cover all the protuberances underneath to form a smooth lubricant layer.

The surface wettability of as-prepared coatings was demonstrated by the static contact angle (CA). As seen in Figure 4D, compared with the intrinsic hydrophilicity of bare Al (CA = 76.2 ± 1.9°), excellent superhydrophobicity was obtained on the surfaces of HN1-1 and SN1-1 with the CAs up to 157.9 ± 1.7° and 149.1 ± 1.8°, respectively. This was mainly due to the synergistic effect of the microscopic rough structure and low-surface-energy material functionalized with modified SiO_2_ NPs, which enabled the SHS to trap a large amount of air, further generating the water-repellent effect. After infusion with lubricants, the fabricated SLIPS exhibited CA decreasing to 113 ± 2.2° and 95.7 ± 1.4°, respectively, indicating the lubricants had been effectively encapsulated in the porous micro-/nanostructure, and capillary force and van der Waals force mainly accounted for the strong adhering strength of lubricant.^48^

### 3.2 Anti-Frosting Capacity

#### 3.2.1 Frosting Delay Time

**Figure 5:**
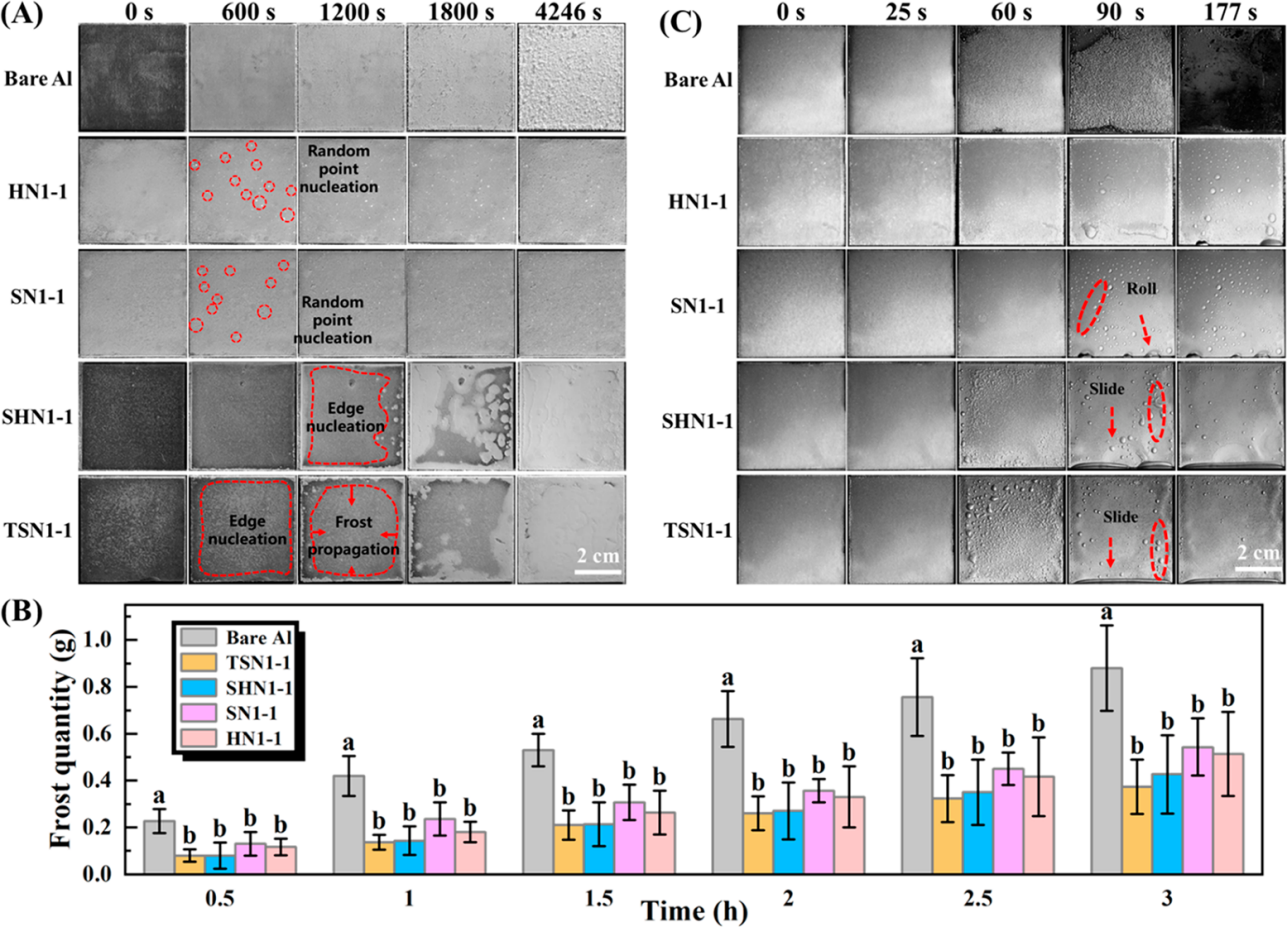
(A) Images of frosting on bare Al and the prepared SHS and SLIPS under extreme conditions (−15 °C, 55% RH), (B) frosting quantity on bare Al and prepared SHS and SLIPS at −15 °C for 3 h, and (C) defrosting process on vertical placed bare Al and the prepared SHS and SLIPS under a room temperature of 20 °C.

**Figure 6:**
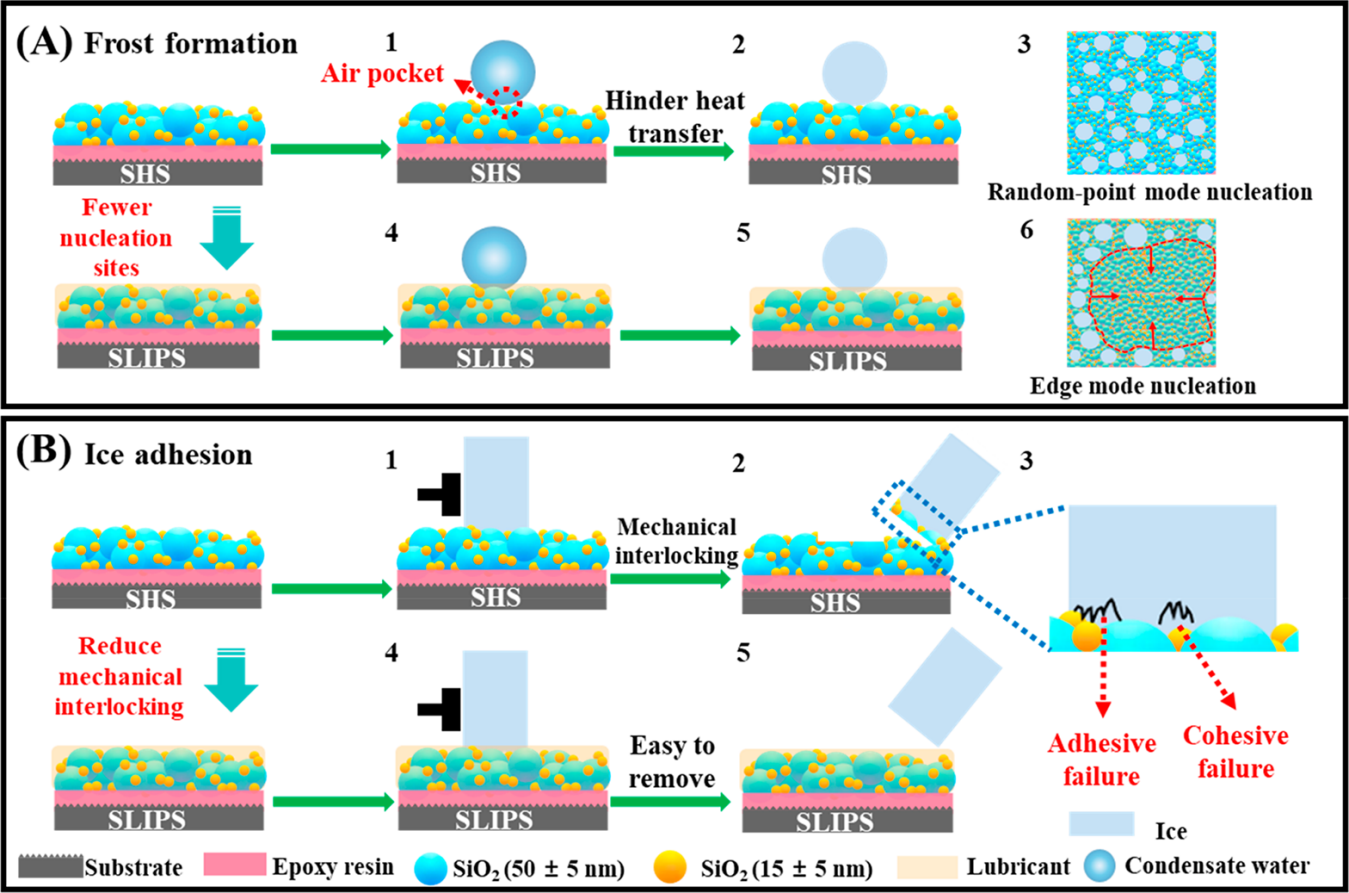
Schematic illustrations of the anti-icing mechanisms of SHS and SLIPS. (A) Frost formation. 1- Water vapor condensed on SHS. 2- Water droplets frozen on SHS. 3- Frost formation on SHS from the top view. 4- Water vapor condensed on SLIPS. 5- Water droplets frozen on SLIPS. 6- Frost formation on SLIPS from the top view. (B) Ice adhesion. 1- The plastic cuvettes with water were inverted on SHS. 2- Icicle was removed from the SHS. 3- Magnification of icicle frozen on SHS. 4- The plastic cuvettes with water were inverted on SLIPS. 5- Icicle was removed from the SLIPS. The ice adhesion strength was tested after freezing at −15 °C for 3 h.

The frost formation process of bare Al and treated surfaces over time was simulated, and results are presented in Figure 5A. The frost formation on bare Al was very soon, and there was a thick frost layer formed on it within 600 s, showing that the bare Al surface was easily wetted by condensed water under the same low-temperature and high-humidity environment (−15 °C, 55% RH) due to its intrinsic hydrophilicity (Figure 4D). By contrast, scattered water droplets condensed on HN1-1 and SN1-1 and formed random nucleation points (Figures 5A and 6A) in 600 s. As time went on, more water vapor coagulated on the two SHS to form large “Cassie droplets” and gradually turned into successive frost layers after 1800 s (Movie S1). The above results could be attributed to the different ice crystal growth modes on hydrophilic and hydrophobic surfaces, which are along-surface and off-surface growth modes, respectively.^49,50^ And, the air trapped in the micro-/nanostructure prevented water from contacting the concave of the surface so that the condensate could only stay on the top of the microconvex structure (Figure 6A). According to the classical nucleation theory, heterogeneous nucleation is more difficult to occur on convex surfaces than on smooth or concave surfaces,^51^ delaying the frosting of the prepared SHS.

However, there was only some frost formation on the edges of the two SLIPS within 1200 s (Figure 5A). The condensate droplets floated on the lubricant layer but not penetrated the void of rough surfaces as shown in Figure 6A, which was consistent with the result reported by Wei et al.^35^ The frost developed from edge to middle on the SLIPS (Figure 5A), being quite different from bare Al, SN1-1, and HN1-1. Ultimately, a loose ice layer formed on SLIPS after 4246 s. The main reason was that the silicone oil and camellia seed oil with low freezing point infused in SLIPS could furnish a decreased ice-surface contact area, possessing an ultrasmooth solid–liquid interface, forming fewer pinning points than the solid–solid interface of the SHS. These results suggest that the prepared SLIPS show a superior anti-frosting property.

#### 3.2.2 Frosting Quantity Test

Figure 5B shows the frosting quantity of bare Al and prepared coatings with time under the same temperature. It was observed that the frosting amounts of SHS and SLIPS were significantly less than that on the bare Al surface (*p* < 0.05) at the same time, but there was no significant difference between SHS and SLIPS (*p* > 0.05), which was mainly due to the frosting delay effect on the SHS and SLIPS in the early stage. With the extension of time, the superwettability gradually became ineffective and condensed water began to aggregate on the prepared SHS and SLIPS. However, the final frost quantity on the bare Al was more than 2 times that of on TSN1-1 and SHN1-1 after 3 h. This could be attributed to the fact that the smooth surface of SLIPS decreased heterogeneous nucleation sites, which effectively inhibited the formation and accumulation of frost crystals and further reduced the speed of frost crystal propagation, showing great frost resistance.

#### 3.2.3 Defrosting Process Observation

Finally, the defrosting process of bare Al and tested surfaces was observed at a room temperature of 20 °C after completely frosting for 3 h. As seen in Figure 5C, it took nearly 60 s for the bare Al surface to start melting (Movie S2), and the defrosting water stuck tightly to the surface, forming a large area of water membrane after 177 s. Ma et al. also mentioned a similar phenomenon, pointing out that defrosting water on bare metal was difficult to be removed by natural force, and could only be evaporated by high-temperature heating.^52^ While the defrosting water on the SN1-1 and HN1-1 shrunk instantly and formed dense small droplets in 60 s, these droplets merged and aggregated quickly by low-surface-energy substances, which could be rolled naturally by gravity (Figure 5C) or easily removed from the surface by a small force.^49^ It was reasonable to speculate that the SHS were not completely wetted due to the air “pocket” effect during the frosting, and most of the frost layer floated on the top of the rough structure in a Cassie-ice state (Figure 6A). In contrast, the frost layer on the SLIPS began to melt in just 25 s (Movie S2), and the melting water could slide directly from the surface under the action of lubricant to avoid its secondary freezing.^35^ Ultimately, the melting water retention of SLIPS was significantly less than that of bare Al and SHS surfaces, exhibiting rapid and excellent defrosting properties.

### 3.3 Ice Phobic Property

As superwetted surfaces with anti-frosting properties could not completely prevent ice formation when exposed to extremely low temperatures for a long period of time, for a systematic evaluation of the anti-freezing capacity on a surface, the ice phobic property should be considered, which reflects the interfacial ice adhesion after the surface is covered by ice.

#### 3.3.1 Ice Adhesion Strength

**Figure 7:**
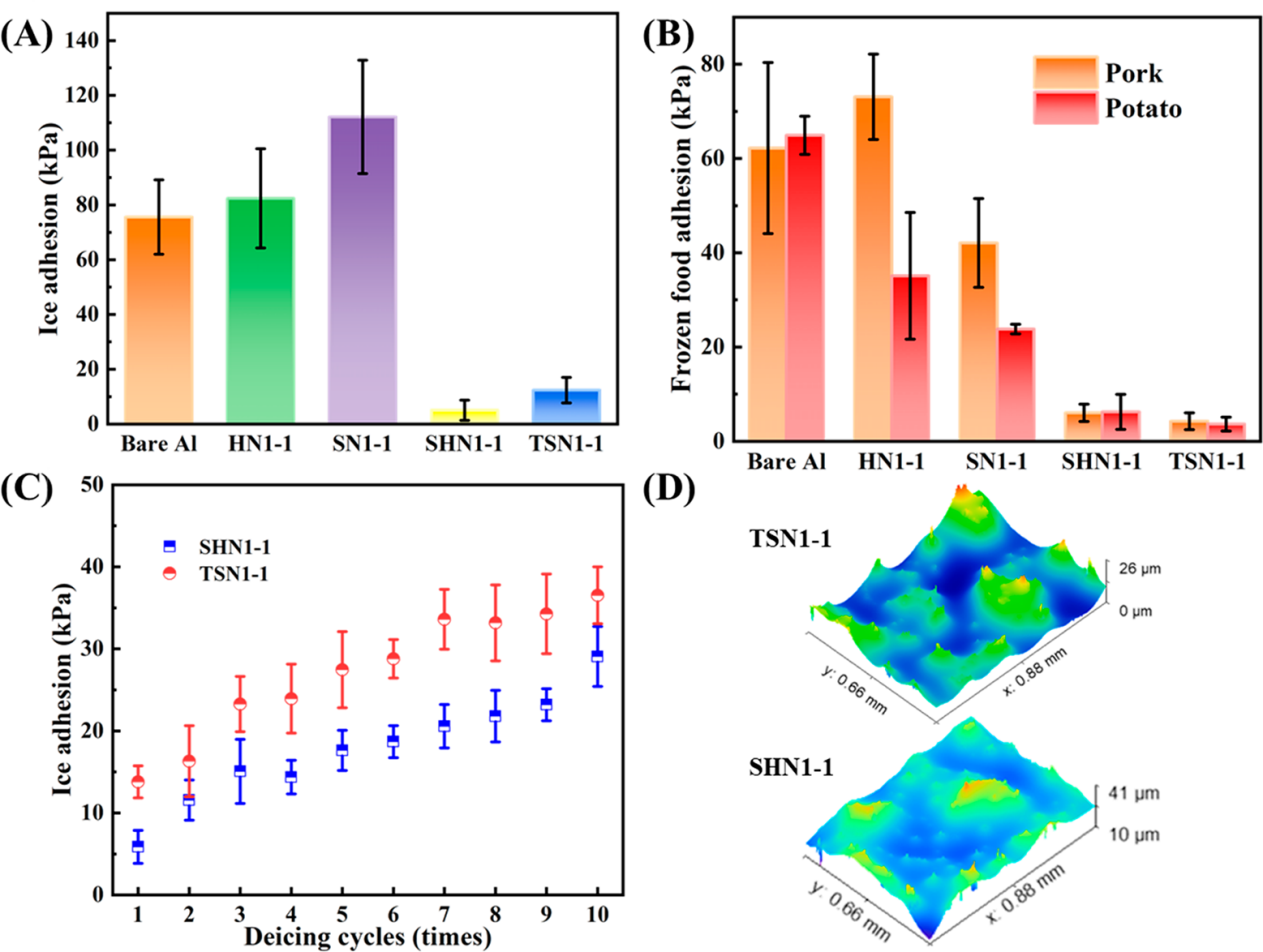
Measurement of ice adhesion strength. (A) Ice adhesion strength and (B) the adhesion strength of frozen pork and potato on bare Al and the prepared SHS and SLIPS. (C) Ice adhesion strength of HN1-1 and SN1-1 after icing/deicing cycles. (D) 3D surface profiles of HN1-1 and SN1-1 after final deicing cycles.

Using the ice adhesion testing platform shown in Figure 1C, the ice adhesion strengths (τ) on bare Al and prepared surfaces were measured. As shown in Figure 7A, the ice adhesion strengths of SHS (τ_HN1-1_ = 82.4 ± 18.1 kPa, τ_SN1-1_ = 112.1 ± 20.7 kPa) were even higher than that of bare Al (75.6 ± 13.6 kPa), which was contrary to some literature showing that SHS weakened the ice adhesion.^51,53^ The reason was that the water would penetrate into the porous structure of SHS under the action of supercooling and hydrostatic pressure, and form strong mechanical interlocking with the micro-/nanostructure including the adhesive strength between the ice and substrate as well as the cohesive strength when freezing (Figure 6B), causing a larger solid-ice contact area and ice adhesion force,^23^ which needed to overcome much more external force (Figure 6B). However, the lubricants infused in the rough structure blocked the direct contact between the ice and the porous structure (Figure 6B) during freezing, and the ice adhesion strengths of SLIPS (τ_SHN1-1_ = 5.0 ± 3.7 kPa, τ_TSN1-1_ = 12.4 ± 4.7 kPa) were 6 times lower than that of bare Al (τ_Bare Al_ = 75.6 ± 13.6 kPa). Particularly, the SA-modified porous surface TSN1-1 with edible camellia seed oil generated an ice adhesion strength as low as 12.4 ± 4.7 kPa (Figure 7A), which might facilitate the design and development of eco-friendly and food-safe SLIPS in the food industry such as for food packaging and other food cold contact surfaces.

#### 3.3.2 Frozen Food Adhesion Strength

In the practical production and storage process, frozen food often sticks tightly to the packaging or conveyor belts, resulting in quality destruction. Based on the above results of ultralow ice adhesion, it is hoped that the prepared SLIPS could be efficient for interfacial anti-freezing during frozen food production. Therefore, the ice adhesion strengths of frozen pork and potato on SLIPS were investigated. As seen in Figure 7B, the ice adhesion strengths of frozen pork and potato from SHN1-1 and TSN1-1 were both less than 10 kPa, which were 1 order of magnitude lower than that on bare Al (τ_pork_ = 62.2 ± 18.14 kPa, τ_potato_ = 64.9 ± 4.05 kPa), announcing great potentials of SLIPS as interfacial anti-freezing materials. It could be visibly seen that the pork and potatoes possessed less adhesion strength on the prepared SLIPS than that of icicles (Figure 7A). The reason was that protein fibers in pork and starch in potatoes and other components have a certain water-holding capacity, affecting the internal migration of moisture out of food. In addition, grease contained in pork can also play a lubricating effect, so, theoretically, separating frozen food from the cold surface would be easier than removing the same volume of ice.

#### 3.3.3 Deicing Durability of SLIPS

It was evident from the frosting delay and ice adhesion strength tests that the anti-icing capacities of SLIPS were superior to SHS. To further evaluate the durability of the SLIPS, icing/deicing cycles were conducted on TSN1-1 and SHN1-1. Ice columns with a cross-section of 1 cm × 1 cm were frozen and separated 10 times at the same position on the same surface. After 10 deicing cycles, the ice adhesion strength on the TSN1-1 and SHN1-1 increased to 36.5 ± 3.5 and 29.1 ± 3.7 kPa (Figure 7C), respectively, showing an increasing trend, mainly caused by the loss of the lubrication layer on the surfaces during repeated shearing, exposing the micro/nano rough structure of SLIPS (Figure 7D). However, the ice adhesion strength on the SLIPS was still half lower than that of bare Al (75.57 ± 13.6 kPa). Besides, TSN1-1 and SHN1-1 were comparable with ice-phobic SLIPS reported in the literature as listed in Table 1, indicating that both prepared SLIPS exhibited superior deicing durability.

## 4 Conclusions

In the current study, two slippery liquid-infused porous surfaces (SLIPS) were studied to explore potential interfacial anti-freezing materials in the food freezing-related facility surfaces. The SLIPS were fabricated by spraying modified H-SiO_2_ NPs and S-SiO_2_ NPs suspensions onto Al substrate coated with epoxy resin to obtain superhydrophobic surfaces and then infusing food-safe silicone and camellia seed oils into the SHS. The frosting delay time and ice adhesion strength of SLIPS were both superior to those of SHS and bare Al, suggesting excellent anti-icing capacities of SLIPS. The ice adhesion applications in the frozen pork and potato were significantly decreased to less than 10 kPa, which was 1 order of magnitude lower than that on bare Al. After 10 icing/deicing cycles, the ice adhesion of SLIPS was still half lower than bare Al, demonstrating sustainable durability. This work demonstrates the great potential of using the SLIPS as a green and safe anti-freezing strategy for the frozen food industry.

## Supporting Information Available

Movie of frosting process on bare Al and the prepared SHS and SLIPS under extreme conditions (−15 °C, 55% RH) (MP4)
Movie of defrosting process on vertically placed bare Al and the prepared SHS and SLIPS under room temperature of 20 °C (MP4)
